# Phenolic Compounds from the Leaves of *Castanopsis fargesii*

**DOI:** 10.3390/molecules22010162

**Published:** 2017-01-19

**Authors:** Yong-Lin Huang, Ya-Feng Wang, Jin-Lei Liu, Lei Wang, Takashi Tanaka, Yue-Yuan Chen, Feng-Lai Lu, Dian-Peng Li

**Affiliations:** 1 Guangxi Key Laboratory of Functional Phytochemicals Research and Utilization, Guangxi Institute of Botany, Guangxi Zhuang Autonomous Region and Chinese Academy of Sciences, Guilin 541006, China; wyf@gxib.cn (Y.-F.W.); ljl@gxib.cn (J.-L.L.); jgswl@gxib.cn (L.W.); chyy@gxib.cn (Y.-Y.C.); lufenglai@gxib.cn (F.-L.L.); ldp@gxib.cn (D.-P.L.); 2 Department of Natural Product Chemistry, Graduate School of Biomedical Sciences, Nagasaki University, 1-14 Bunkyo-machi, Nagasaki 852-8521, Japan; t-tanaka@nagasaki-u.ac.jp

**Keywords:** *Castanopsis fargesii*, *Castanopsis*, phenolic, cytotoxicity

## Abstract

In the course of a phytochemical and chemotaxonomical investigation of *Castanopsis* species (Fagaceae), three new phenolic compounds, (3*R*,1′*S*)-[1′-(6″-*O*-galloyl-β-d-gluco-pyranosyl)oxyethyl]-3-hydroxy-dihydrofuran-2(3*H*)-one (**1**), (2*R*,3*S*)-2-[2′-(galloyl)oxyethyl]-dihydroxybutanoic acid (**2**), and (3*S*,4*S*)-3-hydroxymethyl-3,4-dihydro-5,6,7-trihydroxy-4-(4′-hydroxy-3′-methoxyphenyl)-1*H*-[2]-benzopyran-1-one (**3**) were isolated from the fresh leaves of *Castanopsis fargesii*. In addition, a known phenolic glycoside, gentisic acid 5-*O*-α-l-rhamnopyranosyl-(1→2)-β-d-glucopyranoside (**4**) was also isolated and identified. Their structures were elucidated by means of spectroscopic methods including one- and two-dimensional NMR techniques.

## 1. Introduction

The genus *Castanopsis* belongs to the Fagaceae family and is commonly found in the evergreen forests of East Asia. There are about 120 species of *Castanopsis*, however, the classical plant taxonomy of the species is very complicated and sometimes confusing [1], thus, the application of other auxiliary methods and technologies, such as chemotaxonomy and cytotaxonomy, is necessary to identify species within this genus [2]. Previous phytochemical investigations on the plants of this genus *C. fissa*, *C. cuspidata var. seiboldii*, and *C. hystrix* have led to the isolation of triterpene hexahydroxydiphenoyl (HHDP) esters, HHDP glucoses, galloyl, acylated quinic acids, phenol glucosides, condensed tannins, and flavonol glycosides [3,4,5,6,7,8]. 

In this study, we investigated *C. fargesii*, which is widely distributed in southern China, where it is usually used as a traditional medicine for the treatment of diarrhea, hemorrhage, and chronic ulcers [1]. Our preliminary analysis by HPLC and TLC indicated that the leaves are rich in tannins. Subsequent chromatographic separation of the extract yielded two metabolites **1** and **2**, which were identified as 2,3-dihydroxy-2-(2-hydroxyethyl)-butanoic acid derivatives, and two phenolic compounds **3** and **4**. This paper reports the isolation and structural characterization of the new compounds **1**–**3** and an assessment of the cytotoxicity of these molecules.

## 2. Results and Discussion

**Figure 1:**
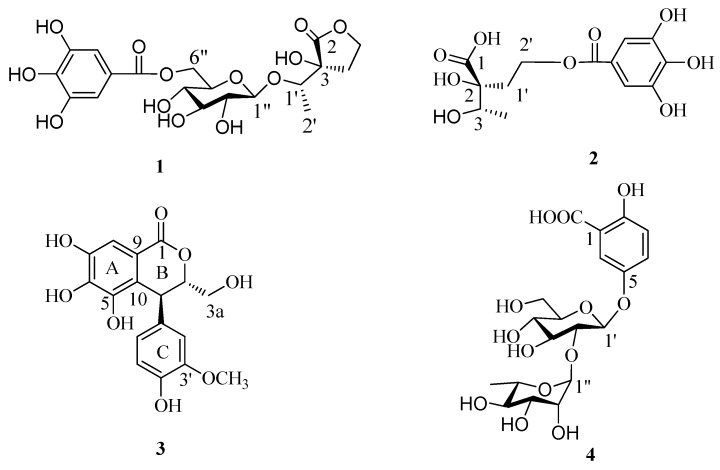
Structures of compounds **1**–**4**.

The fresh leaves of *C. fargesii* were extracted with 80% aqueous ethanol, and the extract was partitioned between Et_2_O and water. The Et_2_O and aqueous fractions were separated by a combination of Sephadex LH-20, MCI gel CHP 20P, Toyopearl Butyl-650C, Chromatorex ODS, and Diaion HP20SS column chromatography and semi-preparative reverse-phase HPLC, to yield three new compounds **1**–**3** and one known phenolic compound gentisic acid 5-*O*-α-l-rhamnopyranosyl-(1→2)-β-d-glucoside [9] (**4**) (Figure 1).

**Figure 2:**
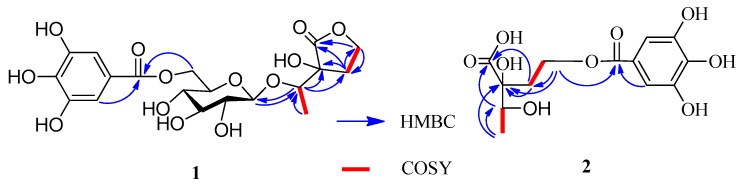
Key HMBC and ^1^H-^1^H COSY correlations of **1** and **2**.

Compound **1** was isolated as a brown amorphous powder and gave a positive FeCl_3_ test (dark blue), which suggested the presence of phenol moieties in the molecule. The molecular formula C_19_H_24_O_13_ was determined based on the liquid chromatography-mass spectrometry IT-TOF (LC-MS/IT-TOF), which showed [M − H]^−^ and [M + Na]^+^ ion peaks at *m*/*z* 459.1143 (calcd. for C_19_H_23_O_13_, 459.1144) and 483.1118 (calcd. for C_19_H_24_O_13_Na, 483.1109), respectively. In the ^1^H- and ^13^C-NMR spectra (Table 1), two proton singlets at δ_H_ 7.13 (2H, s) and four aromatic carbon signals at δ_C_ 109.1 and 145.2, along with an ester carbonyl signal at δ_C_ 166.4 suggested the presence of a galloyl group [10]. The hydrolysis of **1** produced d-glucose, which was identified by GC analysis. The coupling constant of the anomeric sugar proton was 7.8 Hz, indicating that the sugar moiety was in the β configuration. The large downfield shift of the glucose H-6″ protons (δ_H_ 4.32 and 4.58) suggested esterification with the galloyl moiety at this position. This was confirmed by the HMBC correlation between H-6″ and the carboxy carbon (δ_C_ 166.4) (Figure 2). A second HMBC correlation between H-1″ and C-1′ suggested that the glycosyl group is linked to C-1′. HSQC experiment showed that the remaining moiety was composed of six carbons: a carboxy carbon (δ_C_ 178.6, C-2), an oxygenated methine (δ_C_ 77.8, C-1′), an oxygenated quaternary carbon (δ_C_ 76.6, C-3), an oxygenated methylene (δ_C_ 65.8, C-5), a methylene (δ_C_ 29.9, C-4), and a methyl (δ_C_ 15.6, C-2′) carbon. The presence of a γ-lactone structure in the remaining moiety was suggested by the lower field shift of the carboxy carbon (δ_C_ 178.6) and the unsaturation index (eight). This was also confirmed by HMBC correlations shown in Figure 2, in which the oxygenated methylene protons (δ_H_ 4.31–4.34, H-5) correlated with C-2. Furthermore, the ^1^H-^1^H COSY correlations of H-4 (δ_H_ 2.10) with H-5 (δ_H_ 4.32) and H-1′ with H-2′ and the HMBC correlations of H-1′ with C-3 and C-4 and H-4 with C-2, C-3, and C-5 indicated that the remaining moiety is 3-hydroxy-3-(1′-hydroxyethyl)dihydrofuran-2(3*H*)-one. Consequently, the structure of compound **1** was established as 3-[1′-(6″-*O*-galloyl-β-d-glucopyranosyl)oxyethyl]-3-hydroxy-dihydrofuran-2(3*H*)-one.

Compound **2** was isolated as a yellow amorphous powder and gave a positive FeCl_3_ test (dark blue). The presence of a galloyl group was deduced from the ^13^C-NMR signals (Table 1) [6]. The molecular formula of C_13_H_16_O_9_ was established based on the LC-MS/IT-TOF (*m*/*z* 339.0672 [M + Na]^+^, calcd. for C_13_H_16_O_9_Na, 339.0687) and ^13^C-NMR data. The ^13^C-NMR and DEPT spectra showed signals attributable to a methyl (δ_C_ 16.9), a methylene (δ_C_ 34.2), an oxymethine (δ_C_ 71.4), an oxymethylene (δ_C_ 60.2), an oxy quaternary (δ_C_ 77.7), and a carboxyl (δ_C_ 175.4) carbons. The NMR data of **2** (Table 1) were similar to those of **1**, except for the absence of the signals for one glucosyl moiety, which was supported by its MS data. A 2,3-dihydroxy-2-(2′-oxyethyl)-butanoic acid moiety could be constructed by ^1^H-^1^H COSY correlations (Figure 2) of H-4 with H-3 and H-1′a with H-2′a and the HMBC correlations of H-4 with C-3 (δ_C_ 71.4), H-3 with C-1, H-1′ with C-1, and H-2′ with C-2 (Figure 2). The HMBC correlation of H-2′ with the carboxy carbon (δ_C_ 165.9) indicated that the galloyl group was attached to C-2′. Based on these results, the structure of **2** was determined to be 2-[2′-(galloyl)-oxyethyl]-2,3-dihydroxybutanoic acid. 

**Figure 3:**
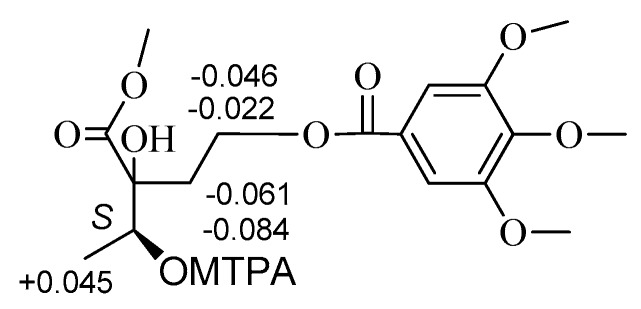
Δδ_(*S*_-*_R_*_)_ values of MTPA ester derivative of **2**.

**Figure 4:**
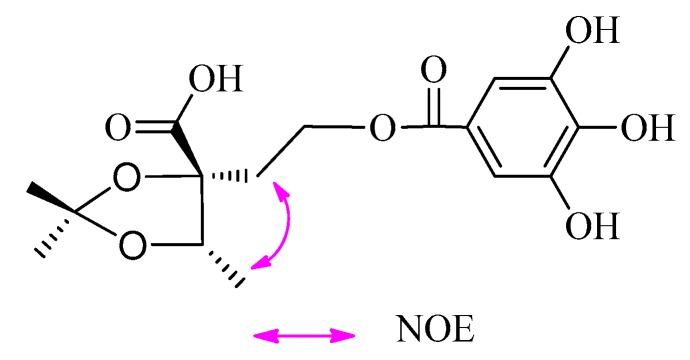
Key NOE correlations of 2,3-*O*-isopropylidene derivative of **2**.

The absolute configurations at C-2 and C-3 of **2** were established using the modified Mosher’s method [11,12]. Treatment of **2** with CH_3_I, then with (*R*)-(−)- and (*S*)-(+)-2-methoxy-2-trifluoromethyl-2-phenylacetyl (MTPA) chloride to get the C-3 (*S*)- and (*R*)-MTPA ester derivatives, respectively. Δδ values obtained from the ^1^H-NMR data of the C-3 (*R*)- and (*S*)-MTPA ester derivative indicated that the absolute configuration at C-3 of **2** was *S* (Figure 3). Compound **2** reacted with 2,2-dimethoxypropane (DMP) and pyridinium *p*-toluene sulfonate (PPTS) to form the 2,3-*O*-isopropylidene derivative. The C-2 and C-3 relative configuration of 2,3-*O*-isopropylidene derivative was determined based on the NOE correlation of H-1′ with H_3_-4 (Figure 4). Thus, the absolute configurations at C-2 and C-3 of **2** were assigned as *R* and *S*, respectively.

Compounds **1** and **2** both contain a 2,3-dihydroxy-2-(2-hydroxyethyl)-butanoic acid moiety. The hydrolysis of **1** in 1 M HCl yielded 3-hydroxy-3-(1-hydroxyethyl)dihydrofuran-2(3*H*)-one that was identified to have the same absolute configuration as **2** by comparing their ${\left[\alpha \right]}_{D}^{25}$ and CD data. Hence, the absolute configurations of **1** were assigned as 3*R*,1′*S*.

**Figure 5:**
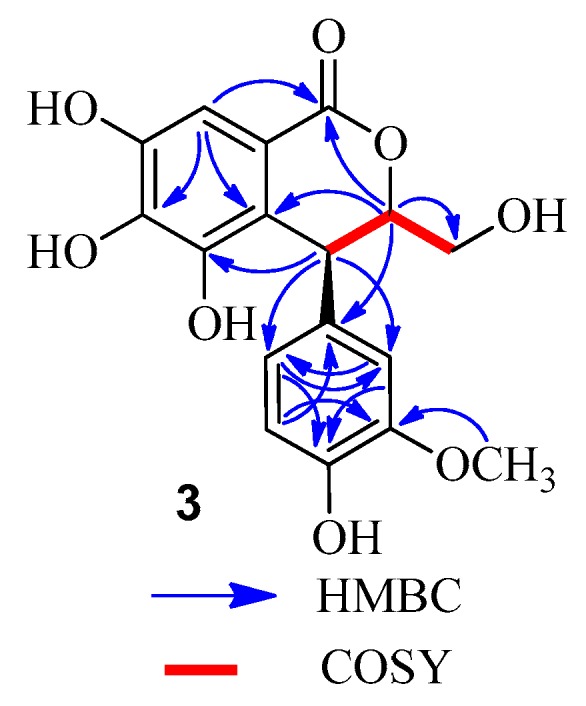
Key HMBC and COSY correlations of **3**.

Compound **3** was obtained as a brown amorphous powder, which gave a dark blue color with FeCl_3_. The molecular formula C_17_H_16_O_8_ was deduced from the [M − H]^−^ peak at *m*/*z* 347.0768 in the LC-MS/IT-TOF (calcd. for C_17_H_15_O_8_, 347.0772). Comparison of the ^1^H- and ^13^C-NMR data of **3** (Table 2) and (3*S*,4*S*)-3-[(β-d-glucopyranosyl)oxymethyl]-3,4-dihydro-5,6,7-trihydroxy-4-(4′-hydroxy-3′-methoxyphenyl)-1*H*-[2]-benzopyran-1-one [13] revealed that the methyl at C-3 in the known compound was replaced by a hydroxymethyl in **3**. This was confirmed by the MS data and the correlations of the methine proton (δ_H_ 4.67) with the methylene carbon (δ_C_ 62.4) in the HMBC spectrum (Figure 5), as well as ^1^H-^1^H COSY correlations of H-3 (δ_H_ 4.67) with H-3a (δ_H_ 3.54) and H-4 (δ_H_ 4.50) (Figure 5). Comparison of the CD and the optical rotation (${\left[\alpha \right]}_{D}^{25}$ +18.3°) data of **3** with those of similar compounds suggested that the absolute configuration is 3*S*,4*S* [14]. Based on the above evidences, the structure of compound **3** was concluded to be (3*S*,4*S*)-3-hydroxymethyl-3,4-dihydro-5,6,7-trihydroxy-4-(4′-hydroxy-3′-methoxyphenyl)-1*H*-[2]-benzopyran-1-one.

All isolates were subjected to a cytotoxicity assay in vitro against human lung epithelial A549, human hepatocellular carcinoma SMMC-7721 cell, human gastric carcinoma MGC-803 cell, liver hepatocellular HepG2 cell, and human breast adenocarcinoma MCF-7 tumour cell. Unfortunately, none of the isolates showed inhibitions of those tumour cells at the highest concentration tested (IC_50_ value > 10 μM).

## 3. Experimental Section

### 3.1. Materials

The leaves of *C. fargesii* were collected at Guangxi Institute of Botany, Guangxi, China, in August 2014, and were identified by Prof. Shi-Hong Lu. A voucher specimen (20140627) was deposited in the Guangxi Key Laboratory of Functional Phytochemicals Research and Utilization, Guangxi Institute of Botany, China.

### 3.2. General Experimental Procedures

Optical rotations were measured with a 341 digital polarimeter (Perkin-Elmer Corp., Waltham, MA, USA). ^1^H- and ^13^C-NMR spectra were measured in acetone at 27 °C, using an Avance 500 spectrometer (500 MHz for ^1^H and 125 MHz for ^13^C, Bruker Biospin AG, Fällanden, Switzerland). Coupling constants and chemical shifts were given in Hz and on a δ (ppm) scale, respectively. GC was performed on a 6890N instrument equipped with a FID detector (Agilent Technologies, Santa Clara, CA, USA) operated at 280 °C (column: 28 m × 0.32 mm i.d. HP-5, column temp. 160 °C). LC-MS/IT-TOF was recorded on a LCMS-IT-TOF spectrometer (Shimadzu, Kyoto, Japan). Semi-preparative HPLC was performed on an Agilent 1200 apparatus equipped with a UV detector and a Zorbax SB-C-18 (9.4 × 250 mm) column (Agilent). Column chromatography (CC) was performed using Sephadex LH-20 (25–100 μm; GE Healthcare Bio-Science AB, Uppsala, Sweden), MCI gel CHP 20P (75–150 μm; Mitsubishi Chemical, Tokyo, Japan), Diaion HP20SS (Mitsubishi Chemical), Chromatorex ODS (100–200 mesh; Fuji Silysia Chemical, Aichi, Japan), and Toyopearl Butyl-650C (TOSOH, Tokyo, Japan) columns. TLC was performed on precoated Kieselgel 60 F_254_ plates (0.2 mm thick; Merck, Darmstadt, Germany) with toluene–HCO_2_Et–HCO_2_H (1:7:1, *v*/*v*) as the solvent, and spots were detected by spraying with a 2% ethanolic FeCl_3_.

### 3.3. Extraction and Separation

The fresh leaves of *C. fargesii* (5.20 kg) were cut into small pieces and extracted three times with EtOH/H_2_O (8:2, *v*/*v*, 36 L) by maceration at room temperature for 7 days. The extracts were combined and concentrated under reduced pressure to give an aqueous solution. The solution was partitioned with Et_2_O four times to give the Et_2_O fraction (32.4 g). The aqueous layer was subjected to Sephadex LH-20 CC (8 cm i.d. × 40 cm) with 0%–100% MeOH–H_2_O (20% stepwise elution, each 1.5 L) to give 9 fractions: frs 1 (15.6 g), 2 (84.5 g), 3 (26.6 g), 4 (27.0 g), 5 (130.0 g), 6 (12.9 g), 7 (3.3 g), 8 (2.3 g), and 9 (2.2 g). Fraction fr. 2 (84.5 g) was separated by MCI gel CHP 20PCC (6 cm i.d. × 40 cm) with MeOH–H_2_O (10% stepwise elution, each 1.0 L) to yield seven fractions, and fraction fr. 2-2 (5.3 g) was further fractionated by Diaion HP20SS CC (4 cm i.d. × 30 cm) with H_2_O containing increasing proportions of MeOH (0%–100%, 10% stepwise elution, each 0.5 L) to give **4** (105 mg). The Et_2_O fraction was subjected to MCI gel CHP 20PCC (5 cm i.d. × 50 cm) with 0%–100% MeOH in H_2_O (10% stepwise elution, each 0.5 L) to yield 10 fractions: frs E-1 (5.3 g), 2 (7.5 g), 3 (1.6 g), 4 (3.2 g), 5 (4.3 g), 6(1.5 g), 7 (2.3 g), 8 (1.0 g), 9 (9.9 g) and 10 (3.5 g). Fr. E-3 was fractionated by Toyopearl Butyl-650C CC (3 cm i.d. × 30 cm) with 0%–100% MeOH–H_2_O containing 0.1% CF_3_CO_2_H (TFA) (10% stepwise elution, each 0.3 L) to give fr. E-31 (1.3 g) and fr. E-32 (122 mg). Fr. E-32 was further purified by Chromatorex ODS CC (3 cm i.d. × 30 cm) with 0%–80% MeOH in H_2_O (5% stepwise elution, each 0.2 L) to give **3** (12 mg). Fraction E-4 was separated by Sephadex LH-20 CC (4 cm i.d. × 40 cm) with H_2_O containing increasing amounts of MeOH (0%–100%, 10% stepwise elution, each 0.5 L) to yield Fr. E-41 (250 mg), Fr. E-42 (150 mg), Fr. E-43 (296 mg) and Fr. E-44 (1.7 g). The Fr. E-42 and Fr. E-43 were further purified by semi-preparative HPLC (MeCN/H_2_O, 20:80, 2.5 mL/min) to give **1** (46 mg, *t*_R_ 14.5 min) and **2** (68 mg, *t*_R_ 13.2 min), respectively.

### 3.4. Spectroscopic Data

*(3R,1'S)-[1'-(6"-O-Galloyl-β-d-glucopyranosyl)oxyethyl]-3-hydroxy-dihydrofuran-2(3H)-one* (**1**): Brown amorphous powder; ${\left[\alpha \right]}_{D}^{25}$ +52.1° (*c* = 0.12, MeOH); UV (MeOH) λ_max_ nm (log ε): 272 (4.32); CD (MeOH) λ_max_ (Δε) 278 (8.4), 254 (4.5), 209 (2.7). ^1^H- and ^13^C-NMR data, see Table 1; LC-MS/IT-TOF *m*/*z* [M − H]^−^ 459.1143 (calcd. for C_19_H_23_O_13_, 459.1144) and [M + Na]^+^ 483.1118 (calcd. for C_19_H_24_O_13_Na, 483.1109). 

*(3R,1'S)-3-Hydroxy-1'-hydroxyethyl-dihydrofuran-2(3H)-one* (*Hydrochloride of* **1**): ${\left[\alpha \right]}_{D}^{25}$ −17.0° (*c* = 0.15, MeOH); UV (MeOH) λ_max_ nm (log ε): 265 (3.16); CD (MeOH) λ_max_ (Δε) 267 (5.7), 251 (3.2), 211 (1.2). ^1^H-NMR (MeOH-*d*_4_, 500 MHz) δ 4.36 (1H, m, H-5a), 4.25 (1H, m, H-5b), 2.11 (1H, m, H-4a), 2.33 (1H, m, H-4b), 1.21 (3H, d, *J* = 6.5 Hz, H-4), 4.12 (1H, q, *J* = 6.5 Hz, H-1′), 1.21 (3H, d, *J* = 6.5 Hz, H-2′); LC-MS/IT-TOF *m*/*z* 169.0475 [M + Na]^+^ (calcd. for C_6_H_1__0_O_4_Na, 169.0477).

*(2R,3S)-2-[2'-(Galloyl)oxyethyl]-dihydroxybutanoic acid * (**2**): Yellow amorphous powder; ${\left[\alpha \right]}_{D}^{25}$ −16.7° (*c* = 0.12, MeOH); UV (MeOH) λ_max_ nm (log ε): 267 (4.26); CD (MeOH) λ_max_ (Δε) 268 (10.6), 252 (4.6), 212 (1.4). ^1^H- and ^13^C-NMR data, see Table 1; LC-MS/IT-TOF *m*/*z* 339.0672 [M + Na]^+^ (calcd. for C_13_H_16_O_9_Na, 339.0687).

*(3S,4S)-3-Hydroxymethyl-3,4-dihydro-5,6,7-trihydroxy-4-(4'-hydroxy-3'-methoxyphenyl)-1H-[2]-benzopyran-1-one* (**3**): Brown amorphous powder; ${\left[\alpha \right]}_{D}^{25}$ +18.3° (*c* = 0.11, MeOH); UV (MeOH) λ_max_ nm (log ε): 220 (4.26), 278 (2.35); CD (MeOH) λ_max_ (Δε) 288 (11.4), 242 (6.5), 218 (2.7). ^1^H- and ^13^C-NMR data, see Table 2; LC-MS/IT-TOF *m*/*z* 347.0768 [M − H]^−^ (calcd. for C_17_H_15_O_8_, 347.0772).

### 3.5. Preparation of MTPA Esters Derivatives 

CH_3_I (30 mg) and K_2_CO_3_ (15 mg) were added to a solution of 2 (10 mg) in DMF (5 mL). After stirring for 24 h at room temperature (r.t.), the reaction mixture was suspended in H_2_O and extracted with CHCl_3_. The CHCl_3_ layer was vacuum dried to afford a residue (6.2 mg). Then, DMAP (3.8 mg), Et_3_N (4.0 μL), and (*R*)-(−)-MTPACl (3.0 μL) were added to a solution of the residue (3.1 mg) in CH_2_Cl_2_ (1.0 mL) and stirred for 4 h at r.t. The reaction mixture was dried under a stream of N_2_. Separation of the residue was done by a silica gel column (hexane/EtOAc, 4:1) to afford the (*S*)-MTPA ester derivative (2.1 mg). The (*R*)-MTPA ester derivative (2.3 mg) was obtained according to the same procedure using (*S*)-(+) MTPACl. 

(3*S*)-*MTPA Ester derivative of* **2**: Colorless oil; ^1^H-NMR (MeOH-*d*_4_, 500 MHz) δ 7.0512–7.3901 (7H), 4.8212 (1H, q, *J* = 6.6 Hz, H-3), 1.3648 (3H, d, *J* = 6.6 Hz, H-4), 2.2511 (1H, m, H-1′a), 2.3004 (1H, m, H-1′b), 4.2526 (1H, m, H-2′a), 4.2812 (1H, m, H-2′b), 3.3206–3.8516 (-OCH_3_ × 5). LC-MS/IT-TOF *m*/*z* 611.17147 [M + Na]^+^ (calcd. for C_27_H_31_F_3_O_11_Na, 611.17162).

(3*R*)-*MTPA Ester derivative of* **2**: Colorless oil; ^1^H-NMR (MeOH-*d*_4_, 500 MHz) δ 7.0510–7.3902 (7H), 4.8210 (1H, q, *J* = 6.6 Hz, H-3), 1.3603 (3H, d, *J* = 6.6 Hz, H-4), 2.2572 (1H, m, H-1′a), 2.3088 (1H, m, H-1′b), 4.2548 (1H, m, H-2′a), 4.2858 (1H, m, H-2′b), 3.3312–3.8716 (-OCH_3_ × 5). LC-MS/IT-TOF *m*/*z* 611.1710 [M + Na]^+^ (calcd. for C_27_H_31_F_3_O_11_Na, 611.1716).

### 3.6. Preparation of Acetonide Derivative of ***2***

Compound **2** (10.2 mg) was dissolved in acetone (1.0 mL) and treated with DMP (0.2 mL) and PPTS (6.5 mg) at r.t. After 4 h, Et_3_N (7.5 μL) was added and the mixture was concentrated by N_2_ blowing. The residue was separated on a silica gel column (CH_2_Cl_2_/EtOAc, 4:1–2:1) to afford the 2,3-*O*-isopropylidene derivative (3.2 mg) of **2**.

*2,3-O-Isopropylidene Derivative of* **2**: Colorless oil; ^1^H-NMR (MeOH-*d*_4_, 500 MHz) δ 6.98 (2H, s), 4.36 (1H, q, *J* = 6.5 Hz, H-3), 1.21 (3H, d, *J* = 6.5 Hz, H-4), 2.11 (1H, m, H-1′a), 2.23 (1H, m, H-1′b), 4.25 (1H, m, H-2′a), 4.29 (1H, m, H-2′b), 1.27 (3H, s, acetonide-CH_3_), 1.31 (3H, s, acetonide-CH_3_); LC-MS/IT-TOF *m*/*z* 379.1007 [M + Na]^+^ (calcd. for C_18_H_20_O_9_Na, 379.1005).

### 3.7. Acid Hydrolysis and Sugar Analysis by GC 

Compound **1** (6 mg) was dissolved in MeOH (4.0 mL) and 1 M H_2_SO_4_ (2.0 mL) and refluxed for 2 h on a H_2_O bath. After the hydrolysate was cool, H_2_O (8.0 mL) was added, then extracted with EtOAc (3 × 10.0 mL). The EtOAc layer was vacuum dried and chromatographed on semi-preparative HPLC eluting with a gradient of MeOH–H_2_O (5:95–25:75, *v*/*v*) to afford 3-hydroxy-1′-hydrooxyethyl-dihydrofuran-2(3*H*)-one. The aqueous layer was neutralized with aqueous Ba(OH)_2_ and evaporated under reduced pressure to give a residue. The residue was dissolved in pyridine (100 μL), subsequent treated with 0.1 mL cysteine methyl ester hydrochloride (150 μL; Sigma, St. Louis, MO, USA) and warmed at 60 °C for 1 h, then the trimethysilylation reagent HMDS/TMCS (hexamethyldisilazane/trimethylchlorosilane/pyridine 2:1:10; Acros Organics, Geel, Belgium) was added and warmed at 60 °C for 30 min. The reaction mixture was partitioned between water and hexane. The hexane extract was analyzed by GC [15] (detector temperature: 280 °C; injector temperature: 250 °C; temperature gradient: start at 160 °C, hold for 5 min, increase to 280 °C at 5 °C/min, hold for 10 min). The authentic samples were analyzed in the same way. The *t*_R_ values of d-glucose and l-glucose were 13.25 min and 15.32 min, respectively. The thiazolidine derivatives of the samples were confirmed by comparison with authentic standards.

### 3.8. Cytotoxicity Assay

All isolates were tested for cytotoxicity in vitro against A549, SMMC-7721, MGC-803, HepG2, and MCF-7 tumour cells via the MTT assay [16,17] with hydroxycamptothecine as a positive control.

## 4. Conclusions

In this study, we separated and identified three new compounds **1**–**3** and a known compound **4** from the leaves of *C. fargesii*. The 2,3-dihydroxy-2-(2-hydroxyethyl)-butanoic acid moiety in **1** and **2** is an unusual carboxylic acid in Nature, thus, these compounds might be recognized as chemotaxonomic markers. Our preliminary examination also suggested this plant contains triterpene HHDP esters, which are important chemotaxonomical markers of *Castanopsis* sp.; therefore, further phytochemical investigations of the leaves of *C. fargesii* are in progress.

## Figures and Tables

**Table 1:** ^1^H (500 MHz) and ^13^C (125 MHz) NMR data of compounds **1** and **2** in acetone-*d*_6_.

Positions	1		2	
	^1^H	^13^C	^1^H	^13^C
1				175.4
2		178.6		77.7
3		76.6	3.88 (1H, q, *J* = 6.5 Hz)	71.4
4	2.10 (1H, m)	29.9	1.19 (3H, d, *J* = 6.4 Hz)	16.9
	2.58 (1H, m)			
5	4.31–4.34 (2H, m)	65.8		
1′	4.01 (1H, d, *J* = 6.4 Hz)	77.8	2.31 (1H, m), 2.36 (1H, m)	34.2
2′	1.26 (1H, d, *J* = 6.4 Hz)	15.6	4.31 (1H, m), 4.33 (1H, m)	60.2
1″	4.41 (1H, d, *J* = 7.8 Hz)	104.1		
2″	3.19 (1H, dd, *J* = 7.8, 8.9 Hz)	73.8		
3″	3.43 (1H, t, *J* = 8.9 Hz)	76.4		
4″	3.46 (1H, t, *J* = 8.9 Hz)	70.4		
5″	3.62 (1H, m)	73.9		
	4.32 (1H, m)			
6″	4.58 (1H, dd, *J* = 2.1, 11.8 Hz)	63.8		
Galloyl				
1		120.6		121.0
2,6	7.13 (2H, s)	109.1	7.10 (2H, s)	109.1
3,5		145.2		145.0
4		138.1		137.7
7		166.4		165.9

**Table 2:** ^1^H-NMR (500 MHz) and ^13^C-NMR (125 MHz) data of compound **3** in DMSO-*d*_6_.

Positions	^1^H	^13^C
1		164.6
2		
3	4.67 (1H, td, *J* = 1.1, 6.5 Hz)	84.7
3a	3.54 (1H, dd, *J* = 7.6, 11.2 Hz)	62.4
	3.68 (1H, dd, *J* = 6.5, 11.2 Hz)	
4	4.50 (1H, br s)	36.7
5		144.7
6		139.7
7		145.3
8	7.15 (1H, s)	107.7
9		115.3
10		119.9
1′		133.5
2′	6.83 (1H, d, *J* = 2.1 Hz)	111.8
3′		147.6
4′		144.8
5′	6.67 (1H, d, *J* = 8.1 Hz)	114.9
6′	6.64 (1H, dd, *J* = 2.1, 8.1 Hz)	119.6
OCH_3_	3.72 (3H, s)	55.4
